# “I am still negative”: Female sex workers’ perspectives on uptake and use of daily pre-exposure prophylaxis for HIV prevention in South Africa

**DOI:** 10.1371/journal.pone.0212271

**Published:** 2019-04-09

**Authors:** Robyn Eakle, Rutendo Bothma, Adam Bourne, Sanele Gumede, Keneilwe Motsosi, Helen Rees

**Affiliations:** 1 Wits Reproductive Health and HIV Institute, Faculty of Health Sciences, University of the Witwatersrand, Johannesburg, South Africa; 2 Department Global Health and Development, London School of Hygiene and Tropical Medicine, London, United Kingdom; 3 Australian Research Centre in Sex, Health & Society, La Trobe University, Melbourne, Australia; Hunter College, UNITED STATES

## Abstract

Women remain highly vulnerable to HIV infection in sub-Saharan Africa, with female sex workers (FSWs) facing some of the highest rates of HIV. Oral pre-exposure prophylaxis (PrEP) has the potential to reduce new infections among populations at highest risk and end-user perspectives of actual use in ‘real-world’ settings are critical to informing PrEP implementation. This paper presents findings from serial in-depth interviews (IDIs) conducted with FSW participants during the course of the Treatment And Prevention for Sex workers (TAPS) Demonstration Project in South Africa, exploring the lived experiences and perceptions of taking up and using PrEP. This research provides insight into risks and responsibilities facing FSWs perceived as prominent drivers in taking up and using PrEP, how PrEP was adopted to mitigate risk or ameliorate realities, and the characteristics of PrEP most valued, all of which are critical to consider in scale-up. Overall, distrust in the existence and/or efficacy of PrEP affected the motivation of women to come to the clinic and to maintain use. As one of the first reports of PrEP use among FSWs outside of a clinical trial setting, this research shows that it will be important to ensure accurate, relevant, and widespread messaging in communities to generate demand and support for PrEP.

## Introduction

The efficacy of dual drug combination (tenofovir and emtricitabine) oral pre-exposure prophylaxis (PrEP) for preventing acquisition of HIV has been extensively investigated and reported [1]. PrEP has been incorporated into combined antiretroviral guidelines for prevention and treatment by the World Health Organization (WHO); is featured in HIV prevention guidelines in several countries and regions; and has been approved by regulatory bodies in at least 15 countries [2]. In most countries currently implementing or piloting PrEP, the delivery is focused towards those at highest risk of acquiring HIV, following recommendations from mathematical modelling [3,4] as well as WHO guidelines.

The focus on those at highest risk stems from the relatively high cost of PrEP which would be mitigated by reducing new infections where they are highest, and from the need of these populations for new options to prevent HIV infection. Women in general are vulnerable, and in sub-Saharan Africa constitute 56% of all people living with HIV [5]. Female sex workers (FSWs) are a subset of this population, with some of the highest HIV prevalence and incidence rates [5]. While FSWs were not included in the oral PrEP efficacy studies as a specific focus population among high risk women [6,7], they are now the focus of many implementation studies in various phases of research across the globe[8]. In South Africa, FSWs make up an estimated 91% of the sex worker population [9], and experience HIV prevalence rates as high as 72% (found in the greater Johannesburg area) [10].

PrEP represents a new opportunity for protection among FSWs who often experience difficulties in ensuring condom use. Key factors affecting their vulnerability include ability to negotiate condom use, violence, poverty, and substance use [11–16]. These factors, in addition to the distinctive structural issue of criminalization of sex work in most countries, make FSWs both uniquely suited for PrEP but also potentially harder to reach with interventions. In this regard, ‘real-world’ studies for FSWs thus far have centred on using an implementation science approach to determine how to integrate PrEP into existing service structures. Beyond the practicalities of implementation, however, is the need to better understand FSWs’ individual perspectives of PrEP.

In these “early days” of PrEP introduction, end-user perspectives of actual use in ‘real-world’ settings are critical to the success of PrEP, and will ultimately determine the fate of future programming. The importance of qualitative research “to answer questions about experience, meaning and perspective”[17] from the position of PrEP users to ensure the relevance in intervention design and successful implementation of PrEP cannot be overstated. While quantitative data will help to characterize populations at highest risk, identify priority geographical settings in terms of HIV incidence for PrEP delivery [18], and evaluate the cascade of care from uptake to retention [19], qualitative data will explain why gaps in successful programming exist and point to how to close those gaps [20].

This paper presents findings from serial in-depth interviews (IDIs) conducted with FSW participants during the course of the Treatment And Prevention for Sex workers (TAPS) Demonstration Project in South Africa. This sample represents one of the first set of FSWs to take up PrEP and report on individual perspectives of actual use. The line of inquiry was situated within a holistic approach based on a social-ecological framework, denoting reflections on the real and practical, rather than theoretical or trial-based use [21]. This research provided the opportunity to examine motivations for and barriers to uptake, as well as what the women most valued about their use of PrEP. Such data will help to inform PrEP implementation scale-up, and in particular demand generation efforts, outreach to potential PrEP users, provider training around PrEP management and adherence support and design of differentiated PrEP services ideally integrated into existing platforms.

The overall aim of the interviews was to elicit feedback on the uptake and use of both an early antiretroviral treatment (ART) for HIV-positive and PrEP intervention for HIV-negative FSWs integrated into an existing, comprehensive health programme tailored for sex workers. This paper will specifically explore the lived experiences and perceptions of taking up and using PrEP among FSWs engaged in the TAPS Demonstration Project.

## Methods

This qualitative research was conducted as a component of the TAPS Demonstration Project and included as part of the main study protocol [22]. TAPS was an implementation study designed to assess whether it was feasible, acceptable, safe and cost effective to roll out oral PrEP and early ART as part of an HIV intervention to FSWs in two urban clinics (Johannesburg and Pretoria) in South Africa [22]. TAPS was launched in March 2015 and completed in July 2017. In general, women had to be currently engaged in sex work, not pregnant or having a major health complication (such as multi-drug resistant tuberculosis) at the time of study enrolment, and had to be age 18 or over to be included in TAPS. The study protocol has been published as well as the primary study findings, where further details on participant inclusion and exclusion criteria as well as demographic characteristics for the entire study population can be found [19,22].

A selection of women enrolled in the study was invited to participate in the additional IDI component during their month 1 clinic visit. TAPS participants were selected using a random sampling algorithm set to select a sample of 15% of those enrolled with the aim of enrolling a sample of up to 10%. This was according to the original design which sought a total enrolled cohort of 400 women across two sites. Women who accepted were required to complete an additional informed consent process for participation in the IDIs at the month 1 visit, and were then scheduled for interviews to be held on or around their 3, 6, and 9 month clinic visits. Completed informed consent forms were kept in separate files at the clinic sites in fire-proof, limited access locked cabinets. After each completed IDI, participants were given R50 (~4 USD) as travel money, however there was no other monetary incentive given in connection with TAPS participation.

Initial interview guides were developed based on findings from a systematic review of qualitative evidence on women’s perspectives on use of female-initiated prevention products in sub-Saharan Africa [23,24], as well as results from formative research conducted in preparation for TAPS [25], including focus group discussions with potential users [26]. The premise and structure of the interview guides were based on the Modified Social-Ecological Model (MSEM) designed by Baral et al [27] to facilitate a holistic and broad inquiry. In practice, we took the levels illustrated in the MSEM, and built questions expanding from the individual to the broadest HIV epidemic level. The first interview guide was then adapted slightly for the second round of interviews, based on findings from the first round, as was the guide for the third round. In this way, themes were linked to follow stories of product use over time, but also adapted to changes observed to target relevant topics. In addition to questions about PrEP beliefs and experience, numerous questions were asked relating to sexual partners, both clients and regular or romantic. The gender of such partners was not explicitly asked as a direct question to participants, although no assumptions regarding gender identity were made during questioning.

The overall aim of the serial interview (often called Longitudinal Qualitative Research or LQR [28]) approach was twofold: 1) to see if and/or how women’s realities changed over time with use of PrEP, and 2) whether they may become more open with the interviewer over time and provide richer narratives of their lives than those presented in the first interview, hopefully reducing social desirability bias in responses [29]. While this method has been prevalent in cancer-related research [28], it has more recently been employed in qualitative components of PrEP-related trial research [30]. It should be noted, however, that the change over time is not the primary focus of this particular paper as it would have limited the exploration of themes to those in which change over time rather than allowing for a broader exploration of the data. It would be difficult to fully comprehend the change over time in this research without first looking at broader perspectives and positioning. Instead, this aspect will be examined in further detail in a future publication likely combining PrEP and early ART. Here, changes in perspectives will be presented as they relate to the key emerging themes. IDIs were digitally recorded, conducted in the participant’s preferred language, usually a combination of local vernacular and English, and lasted up to 1.5 hours. Interviews were later translated and transcribed simultaneously by research assistants into English. Interviews were conducted by research assistants (all women) who had been comprehensively trained in qualitative interview skills and had combined experience of this method of more than five years. No one else was present during the interviews.

Analysis of the data began with development of a coding framework created separately by two researchers and then compared. A final framework aligning codes was developed and employed in 100% coding by both researchers of all transcripts. The framework and transcripts were uploaded and coded in QSR NVIVO 10. Each round of interviews was coded in a separate database using the same coding framework.

After coding was complete, the two researchers compared findings and discussed any differences in coding to align results. This process was completed first, before the analysis of the serial aspect of the data. Thematic analysis was led by the first author, using the Braun and Clarke method [31]. This approach involves a six-phase process: familiarisation with the data, generation of initial codes, searching for themes, reviewing themes, defining and naming themes, and finally producing the report. In this regard, we adopted a contextualist position, where the analysis aimed to report on the experiences and realities of the participants as well as how the external social environment influenced how the participants make meaning of their realities in relation to PrEP.

All interviews were included in the analysis, even though there were not complete sets of three rounds for each participant. For those participants with second and third IDIs, change over time was explored using a trajectory approach, chosen since the aim was to take into account “individuals’ experiences over time” [32]. Coded data were compared as associated with the prominent themes emerging from the initial analysis across the time points for each participant. As there were fewer second and third round IDIs and the change over time was confined to the themes included for this analysis, it was possible to employ a simplified matrix approach and compare results worksheets (developed in Microsoft Word) for the three coded databases by hand. This was conducted independently by the first and second authors, and then findings were compared and found to have few discrepancies which were resolved.

### Ethical considerations

All women participating in the TAPS Demonstration Project completed written informed consent. Those women who participated in the qualitative research component also completed a separate written informed consent for this specific research. The entire TAPS study was reviewed and approved by the Wits Human Research Ethics Committee (reference number: 140502), and the IDI component was also reviewed and approved as part of a PhD project by the London School of Hygiene and Tropical Medicine (reference number 10102). In addition, following any reports of violence during the course of these IDIs, participants were offered relevant assistance, medical or otherwise, as needed. Women’s names were changed to pseudonyms in the text to protect their identities.

## Results

From those invited across the two sites, 18 women from the PrEP arm agreed to participate in the IDIs, 11 in Johannesburg and 7 in Pretoria. Following the first round, 10 women completed the second round of IDIs (4 in Johannesburg, 6 in Pretoria), and 6 completed the third round (1 in Johannesburg, 5 in Pretoria). This dropout relates to the overall rates of loss to follow up across the larger TAPS study [19]. In total, data from 34 interviews were coded and are included within this analysis. IDI participant demographics are shown below in Table 1.

**Table 1:** Qualitative research participant demographics.

Characteristic	Total
**Age**	
23–30	10
31–40	8
**Relationship status**	
Single	8
Steady partner, not married and not living together	6
Not married but living with a partner	3
Chose not to answer	1
**Education**	
Primary	3
Secondary	8
Matric	7
**Place of Birth**	
Gauteng	2
Other SA Provinces*	3
Lesotho	1
Zimbabwe	12
**Place of work**	
Hotel/Brothel	12
Street	5
Other (bush)	1

*Mpumalanga; Eastern Cape

The ages of the PrEP IDI participants ranged between 23 and 40, with 10 between the ages of 23–30, and 8 between the ages of 31–40. None of the women were married, though nine of them had a steady partner, three of whom lived with their partners. A majority of the women were originally from Zimbabwe (n = 12), one from Lesotho, and the rest from Gauteng, Mpumalanga, and Eastern Cape provinces in South Africa. Most of the women had at least some high school education, with three only having completed primary school. Finally, most of the women worked in brothels (n = 12), while the rest worked on the street or in other less formal settings such as vacant lots.

At the start of the project, few of the women had heard about PrEP prior to learning about it during the peer educator outreach and recruitment efforts. With this in mind, the IDIs aimed to situate PrEP within women’s lives, including every day worries, relationships with family, friends, and partners, caring for children, and navigating their working situations. The data presented here fall into themes describing their perceptions of risks and responsibilities, adoption of PrEP, experiences of PrEP use, and interactions with people in their lives as related to PrEP. The themes inherently overlap and are intertwined, according to the complexities and intermingling of relationships and social dynamics of women’s lives interacting with HIV prevention, and PrEP in particular [33]. These data reflect both a practical implementation (or public health) perspective of services and lived experiences. Each participant quote is labelled with a pseudonym, the location (JHB = Johannesburg; PTA = Pretoria), and the IDI number (e.g. first, second, third round).

### Positioning PrEP in the wider context of women’s lives: Risks and responsibilities

Each IDI began by exploring women’s personal realities: how they were feeling, worries and concerns, and everyday meaningful experiences beyond HIV or safer sex. It is within this broad context that PrEP was considered and enacted. Emerging as one of the strongest themes among and across the interviews was women’s articulations of risk and personal responsibilities. Risks arose from their jobs in sex work where STIs and HIV were common, as well as violence experienced both in sex work and the inner cities in which they lived. There were also risks from main partners where condom use was limited. Responsibilities centred on money, needing income to support themselves and family members, as well as pursuing possibilities outside of sex work, to improve their lives.

In one way or another, risks and responsibilities were universal, in particular around sex work: *“No I worry about the life that I’m living …*. *that until when will I be living this life of this job*, *if I could just provide for my children and I just pray that if I can get a job so that I can live a normal life as other people” (Maggie*, *JHB*, *IDI 1)*. The burden of the job and its risks compared with the need to make money was a daily personal conflict: *“Yeah it was like something that is torturing me every day*. *And if I’m doing it*, *I will be telling myself I am digging my own grave here” (Nancy*, *JHB*, *IDI 1)*.

As reflected in their demographic descriptions and voiced in the IDIs, most of the women did not have a sufficient level of education nor skills to apply for other jobs. Some were attending school during the time of the project and were supporting themselves and family members through sex work. *“I couldn’t study because of the baby …*. *I had to run away from them* [school] *because they kept asking me questions*. *There is a teacher who I told because she had seen me by the street and I had to explain it to her why I did what I was doing” (Polly*, *JHB*, *IDI 3)*. Polly acknowledged through the course of her interviews her desire for an education which conflicted with the necessity of working to provide for her daughter, as well as with her ability to come to the clinic. Her story represented a conflicting dynamic common to many of the women where they wanted and needed to continue engagement in care but personal responsibilities made this difficult. In her final interview she articulated her specific desire around future employment: *“I wanted to be a nurse but at the moment I can’t” (Polly*, *JHB*, *IDI 3)*. While none of the women in these interviews had moved to new jobs during this project, over the course of the IDIs ideas for the future became more tangible.

Many of the women were the sole providers for their families and felt the daily stress of supporting them resting heavily on their shoulders: *“Mostly the main thing which is worries me is my kids*, *that is the most worrying*. *Me at home*, *that I must fix everything no matter even to do this situation of work is because of the family thing*, *like my kids and my mom and my dad” (Brenda*, *PTA*, *IDI 1)*.

The burden of these family responsibilities meant that women wanted to remain healthy and recognized their risks of becoming HIV infected, the latter often a key factor in participants choosing to take up PrEP: *“A month I was only telling myself I will go*, *I will go*, *cause I will be busy*. *Jah I just told myself I'll go*, *I'll go next week*, *I'll go tomorrow …*. *Other people are afraid to hear their statuses mostly” (Mbali*, *JHB*, *IDI 2)*. The fear of a potential HIV status may have delayed interest in and initiation of PrEP for many of the participants, but it also seemed to motivate towards a tipping point of interest for those who joined the study and took up PrEP.

When asked about their main motivations for taking PrEP, almost all of them said the same thing—that it was to protect their health given the risks of their work: *“I am looking at the job that I am working its dangerous and it can give me diseases whereas I know that I am not sick” (Thembi*, *PTA*, *IDI 1)*.

There was continuing underlying concern for the potential of HIV infection among these women, which remained even when using condoms and in their initial months of using PrEP. This was a pervasive sentiment. Condom breakage, a common occurrence, was a motivator to take PrEP: *“I was so happy and wanted to join* [TAPS] *immediately because I had a problem of condoms breaking*. *So now even so I know that when the condom breaks there is a medication I have taken to prevent HIV infection” (Maggie*, *JHB*, *IDI 1)*.

Condoms could break through the general course of sex, or when men wanted to break them on purpose which was a common experience and an ongoing fear:

“Aaa what I can say about the pill is that the pill is good. It’s ok cause it helps us from getting HIV, cause sometimes you meet very rough clients to an extent that the condom break. Some will be knowing that he is sick, he becomes rough with us because he wants the condom to break. Some guys are very, I don’t know if I can say, they are rough or rude. I don’t know” (Hope, PTA, IDI 1).

Many women described PrEP in such a way that it could be considered a second-line defence, which made them feel safer: *“No it’s the reason to drink prevention* [take PrEP] *because maybe if I wasn’t taking it*, *then some clients bursts the condom and you don’t know what to do*. *So when the condom bursts and the person has a disease you will not get it” (Sisi*, *PTA*, *IDI 3)*.

This added protection also related to sex with main partners, where condoms were not usually employed. Women who acknowledged condom use, or lack thereof, tended to define a romantic relationship versus sex with clients. This held true whether the women’s partners knew they were sex workers or not. Navigating sexual relations with main partners was challenging, but PrEP gave women peace of mind with their partners when they couldn’t be certain that they were the only lover in their partners’ lives. Among women who had main partners (e.g. regular, steady, or non-paying), most admitted that they couldn’t be sure they were in a monogamous relationship. PrEP was there to keep them safe within such relationships where condom use was less common.

### (Dis)believing in a pill to prevent HIV

This theme subdivides into three inter-connected sub-themes, which convey the complexity of PrEP awareness, beliefs and emerging confidence in the technology.

#### Beliefs about PrEP efficacy

Over and above the motivation to use a product that could help to protect them from HIV and relieve some of the psychological burden in their lives, women needed to grapple with interpreting and understanding the stated efficacy of an entirely new technology. Given several decades of messaging that condoms were the only way to prevent HIV transmission during sexual intercourse, it was not surprising that the news of PrEP was sometimes met with disbelief: “*So but on my side I'm still deciding continue taking them or stop*. *I don’t know now*. *Like what I was saying that people they wanna know if these tablets they are working or they are not working*. *I don’t know*. *For that one I've no idea” (Royal*, *JHB*, *IDI 1)*.

Missy related how some of her colleagues did not believe that PrEP was real, and how she and her sisters investigated the PrEP product:

“My younger sisters they do understand it because they even went online and check what is it that I am taking. And my friends, I explained to them. I even told them that it’s coming on public they will take it although I didn’t tell them that now its only sex workers that are taking this thing. I just told them that at the clinic they gave me this” (Missy, PTA, IDI 1).

Reflecting on initial disbelief in PrEP came more often in later interviews. In her third IDI, Brenda reported how she had found colleagues in her room going through her bag. She worried they were stealing her pills, but she later found that they were actually trying to figure out what the pills were and whether they were real. They were all HIV-positive and couldn’t grasp the concept of PrEP:

“It seems like, the way I heard it is, like they were showing everyone ‘do you know how these tablets work?’ And themselves they can’t drink it …. I am still negative. They don’t believe it. They said ‘why you only? Us we are positive”, you see” (Brenda, PTA, IDI 3).

The other women in Brenda’s workplace struggled to accept that, as a sex worker, Brenda could still be HIV-negative, therefore they grappled with the notion that she had been given pills to *prevent* HIV. They expressed genuine disbelief that preventive pills even existed. Brenda admitted herself that she initially came to the TAPS clinic purely out of curiosity, not entirely believing that PrEP was real.

#### Personal and societal awareness of PrEP

This confusion about whether PrEP was real and actually prevented HIV persisted because it was only available to FSWs in special clinics and not widely reported on in the media, as one participant articulated: *“They ask me why it’s not on the TV or on the radio*? *Why is it private*, *it’s secretive*? *Jah they say if your clinic was real” (Nancy*, *JHB*, *IDI 1)*. Some participants described their initial apprehension about potentially being guinea pigs for drug testing, which was usually laid to rest during the first few months of participation. However, interactions in the community were a different story. When participants told others that PrEP was made of ARVs, it immediately signalled illness and HIV:

*“Others were not happy for me they thought it was an ARV*. *And some understood*, *and some didn’t*, *and with my family they are fine*. *I told him* [boyfriend] *but at first he could not understand that*. *I sat him down and explained it to him*, *then he understood but he then asked why is only for women” (Polly*, *JHB*, *IDI 3)*.

Another participant recounted how she asked another organization working in her community about PrEP: *“So …*. *then I was asking her about the PrEP*. *She said they don’t have*. *And they don’t even know about it” (Brenda*, *PTA*, *IDI 3)*. This general lack of knowledge in the community about the existence of PrEP made it more difficult to believe in on a personal level and then to convince others, especially when only provided to a select few, as Polly’s boyfriend questioned in the earlier quote. Indeed, as the IDIs progressed, women suggested that promoting the message of PrEP to the broader community and making it available to others would help with supporting use by reducing the stigma around ARVs as being only for sick people: “*jah because ah the other friend once saw that medication and asked me about it …*. *But she didn’t understand*, *she wanted to say that they are ARV” (Polly*, *JHB*, *IDI 2)*.

#### Emerging PrEP confidence

Reconciling their own and others’ confusion or disbelief around PrEP was observed across the second and third waves of interviews. This process seemed to contribute to a commitment from these participants to take PrEP, and also circled back to the motivation of managing and mitigating risk.

Disbelief in PrEP eventually dissipated for those who continued in the study, and part of eliminating the personal scepticism was by repeated HIV-negative test results. Motivations to test (and maintain PrEP use) were linked to a firm desire to see continued negative results: *“I don’t like it*, *but for the fact that*, *you guys need to see the results so I do it and I know that I am always negative” (Missy*, *PTA*, *IDI 1)*.

Over time, personal belief paved the way for participants to prove to others that PrEP was real, since they were still HIV-negative. This elicited interest in PrEP from people outside of the study: *“Yes*, *friends and neighbours*. *Because I want them to get the same services I got*. *Like my friend she wants to take it and I told her about mine” (Polly*, *JHB*, *IDI 3)*. After using PrEP for some time, women also wanted it for their main partners: *“I want to ask about this PrEP*. *I have a boyfriend*. *Is it possible for him to come and take this PrEP*?*” (Lebo*, *PTA*, *IDI 2)*.

### Finding health, control, and well-being in PrEP

Growing out of the acknowledgement of personal risks and responsibilities, as well as a confirmed belief that PrEP was real and playing a part in keeping women negative, was the notion of health and well-being reinforced by taking PrEP and through continuous engagement in care. This translated into a commitment to health, hope for the future, and a sense of strength and pragmatism in the present in relation to their work. Several women asserted their personal empowerment in taking PrEP, as Nancy told her friends: *“me I’m doing it*, *if you don’t it’s for your own good*. *I am doing this for my own life*. *Not for anyone” (Nancy*, *JHB*, *IDI 1)*.

Regular clinic visits created a sense of assurance: *“No its good coming here ‘cause you will be knowing your health*. *Unlike just sitting*, *you are just sitting you don’t know what is going on*. *At least when you come here you will be knowing at least some” (Royal*, *JHB*, *IDI 1)*. This, along with access to PrEP, created a level of added control in their lives, especially when armed with the knowledge that PrEP was not a forever regimen. This made it easier to rationalise use and was equated with a potential end to sex work: “*We just talk about that one day when we leave sex work we will quit the tablets” (Pinky*, *JHB*, *IDI 1)*.

Practically, women were given the option to cycle on and off PrEP through the course of the study, and they were counselled on if or how they might do so with the support of a clinician. Many women explained how they decided to cycle off on their own, and would come back for retesting to be able to restart PrEP a few months later. Some of these women related how they would take a break when they went back to their home villages or if they stopped sex work for a time, which seemed again to contribute to a discourse of enhanced control that ran through many of the transcripts.

Beyond control specifically, however, taking PrEP emerged from analysis as a source of positivity, stemming from an underlying feeling of safety: “*I feel …*. *happy*. *I feel great taking Truvada*. *I know I am safe taking*” *(Anna*, *JHB*, *IDI 1)*. Healthiness and happiness was important and underscored by all of the participants in one way or another. Safety also denoted trust, often a synonym for control over women’s positions in their realities as sex workers: *“Since I've been using this medication I'm feel myself like I'm healthy …*. *Mmm I'm just happy*, *just makes me happy and jah and I trust myself” (Polly*, *JHB*, *IDI 1)*.

Some women felt that the pill was helping them beyond preventing HIV. In this regard, some of the participants reported previous feelings of illness which had dissipated: *“…what I discover is like my health is like normal*, *normal I don’t feel pain like I’m shivering anytime like what I did last time before*. *Now I’m feeling a good health*, *a normal breathe” (Joy*, *JHB*, *IDI 1)*. They also reported overall relief from less concrete symptoms, which were more akin to general sensations: *“‘Cause sometimes I normally used*, *I used to like sleeping …*. *but now sometimes*, *something is changing in my body*. *I used to feel like weak*, *in the morning when I'm waking up*. *I think it’s helping” (Hilton*, *JHB IDI 1)*.

When discussing how PrEP made them feel about sex, most women talked about the feeling of added safety, but not in a way which made them want to have more, or to have condomless sex. Most perceived changes in sex was related to their work. As one participant related:

“According to the rumours, they say when you take TAPS, eeh PrEP or ARV, your inner vagina it becomes more hotter like when mens are penetrating they will say you are hot, you are hot things … like most of my clients they say ‘Yoh! You were so hot!’ So I think that’s what changed. I don’t know because I cannot feel myself inside. No, I actually don’t like sex (Missy, PTA, IDI 1).

Weight gain was a limited “side effect” (later attributed to feelings of well-being and not a clinical side effect), and tended to signify enhanced health and strength: *“Even the weight*, *I feel like it gives me weight when I am looking at myself” (Thembi*, *PTA*, *IDI 1)*. While a small number of participants were not happy with the weight gain, for most who gained weight it was an explicit signifier of health, or a measure of personal fortitude and empowerment: *“Mm I think they [pills] make me strong*. *It’s like now I*, *I am protected a lot since I use them*, *and me I can feel in my body that I am fine” (Lebo*, *PTA*, *IDI 1)*.

Finally, there was a significant sense of altruism and a need to support their community among the participants who recognised value in PrEP and having access to it: “*I wanted this programme to help all of us*, *sex workers” (Anna*, *JHB*, *IDI 1)*. This added to the sentiment of hope and the possibility of improving their perceived and physical positions in their work and life realities. Another participant felt that the act of offering PrEP to FSWs was a sign of change in how sex workers were perceived by wider society:

“…. I feel happy there’s some of the government maybe is take care of people because they love everyone. They can’t choice that this one is prostitute and what. They love us. They put us together as one people because they care. So I have proud that if I feel like I have a problem, I go there and tell them what I feel and I’m negative …. they are preventing us rather than to sick and die, leave our kids (Joy, JHB, IDI1).

### Managing the practical complexities around PrEP use

For PrEP to be effective in women there is a need for high adherence and daily pill taking [34,35]. Though not as prominent a theme in the interviews as compared to the others presented here, the practicalities of managing PrEP use and the activities relating to it were still important to consider. In particular were the practical issues of daily pill taking, managing personal relationships in relation to PrEP use, developing supportive structures, considerations around behavioural disinhibition, and managing the practicalities of clinic attendance.

Developing innovative strategies for pill taking was universal, including stashing a pill or two in a bra or a tissue in case of late or unpredictable nights, setting phone alarms, and placing the pill bottle in a routine location like next to a tooth brush. These were not always perfect. Like others, Mbali admitted to sometimes forgetting: *“…*. *we do forget 'oh today I didn't take my pills*!*’*. *Before I was forgetting*, *I don't wanna lie*. *Now I don't forget”* (Mbali, JHB, IDI 2). Often, participants would admit when they missed doses or ended up sharing with someone else also taking PrEP to top up their stores when they couldn’t make it to the clinic on time:

“We do share I don't wanna lie. You told us not to share, but if we run out we do share. We just told ourself it is one thing. We said how can we not share, but this is one thing. When I run out I can just say give me two. I will be drinking my sister’s” (Mbali, JHB, IDI 2).

Most of the women participating in second and/or third IDIs seemed to be more open or honest about struggles with pill taking, while others described becoming better and finding strategies for consistent pill taking. Some suggested, after taking PrEP for a while, that having an injectable would be better than the pill, to alleviate the burden of daily adherence, most likely related to their experiences with or knowledge of injectable contraception.

One way or another, support systems for pill taking were important in maintaining use. Family, friends, or partners could be a source of support: *“Jah like my sister we do take PrEP together*, *so when she is taking I take also” (Mbali*, *JHB*, *IDI 2)*. Loved ones who were HIV-positive could act as a support system for taking PrEP: *“My sister is positive*. *So she is the one who say to me you must go and take” (Mandy*, *JHB*, *IDI 1)*.

Others ended up losing family, friends, or partners over their participation in the study and PrEP use. This was an unfortunate development, but it also usually resulted in a reaction of asserting a sense of empowerment or control. For instance, Missy decided to stay in the study and lose her boyfriend who would not believe that she was taking ARVs for prevention, rather than give up taking PrEP:

“I don’t have my partner anymore he dumped me because I am taking TAPS/PrEP and he doesn’t even believe this …. He, he dumps me because I am drinking this medication which he thinks I am drinking HIV ARV …. Hee ee I did explain, the thing is he lack information too much” (Missy, PTA, IDI 1).

Disinhibition, or discontinuing condom use while using PrEP, was not reflected among most of the participants. Only one woman admitted to sometimes giving more oral sex without a condom than before she started taking PrEP, and a few women spoke of how they had considered not using condoms on occasion. However, given that most still relied on condoms to prevent pregnancy, most of them were discouraged from condom cessation: *“I was scared because what if I get pregnant*? *So if I get pregnant I put my life risk” (Joy*, *JHB*, *IDI 2)*. Others asserted the importance of maintaining broader sexual health, and highlighted the important, but incomplete, contribution that PrEP made in this regard:

“I worry about my health. Because without my health I will not be able to provide for my family …. but I make sure that I prevent all of them. I prevent STI using condom, and prevent pregnancy again using condom and by eeh mmm using the injection, and I also prevent HIV by using PrEP” (Missy, PTA, IDI 1).

Finally, personal treatment at the clinic made it easier to keep coming: *“I like the fact that when I come to the clinic they tell me I look nice*, *they remember me*, *and they will ask me what month am I here for*. *They will tell me go to this one* [staff member], *go to that one” (Missy*, *PTA*, *IDI 1)*. The feeling of the clinic was a big draw for participants in both locations: *“The study is a very good thing to attend and you feel comfortable and safe” (Maggie*, *JHB*, *IDI 1)*. All participants related how the service at the clinic supported their PrEP use and attendance: *“…*. *because you guys are free*, *you are welcoming and one won’t be shy to express and say what they came to the clinic for” (Joy*, *JHB*, *IDI 2)*.

## Discussion

This is one of the first reports of personal experiences and perspectives of actual use of oral PrEP among a group of FSWs outside of a clinical trial setting. This research provides insight into the risks and responsibilities that women perceive in their lives, the ways in which women adopted PrEP to mitigate their risk or ameliorate realities, and the characteristics of PrEP that they most valued, all of which are critical to consider within the context of implementation.

The findings produced from this research are grounded within the MSEM framework, where personal views and factors interact with and are influenced by social and sexual networks, community, societal and public policy, and the HIV epidemic itself. The components of this framework are visible within the themes where community perceptions of PrEP influenced personal beliefs and experiences of use, or where knowledge of the vast epidemic made it difficult to believe there were still HIV-negative FSWs left to take advantage of new prevention methods. The themes identified in this paper make clear the significant role that family, friends and peers play in shaping perceptions of HIV risk, of the efficacy and potential impact of PrEP and, ultimately, in PrEP uptake. The role of social networks in propagating accurate information about PrEP, and in shaping narratives of PrEP confidence and acceptability should not be underestimated.

Disbelief in the existence and/or efficacy of PrEP was a prominent theme, which according to the IDIs, affected the motivation of women to come to the clinic and also affected women trying to maintain use. When those around users are constantly questioning whether such a preventive product can be real, it may be difficult to continue use. This could speak to reasons for dropout in the larger TAPS study for which the primary, programmatic results have been presented [19]. As PrEP had not been widely introduced as a national policy when these interviews were undertaken, there was no generalised community education about its use which may have contributed to mistrust of the product. This disbelief, or distrust, was also a phenomenon seen in the VOICE-D sub-study and HPTN/ADAPT studies which found scepticism of PrEP efficacy to be a significant barrier to use [36,37]. Both Normalization Theory [38] and the Diffusion of Innovations [39] speak to the imperative of community assimilation of interventions for successful implementation. Indeed, without belief in and demonstration of efficacy on a personal level (e.g. repeated negative HIV tests with PrEP use), little engagement can be expected. This was underscored by the participants through the course of the IDIs, and often raised ethical quandaries around not being able to provide others with PrEP. The clinic did, however, allow and promote couples testing as desired by the participants.

The repeated discourse on disbelief or distrust in the existence and efficacy of PrEP also points to a need for concerted community based messaging beyond target populations. While mathematical modelling has suggested that focused PrEP delivery to those at highest risk will have the greatest impact in the most cost-effective manner [3,4,40], the findings in this study combined with those from VOICE-D and ADAPT, imply that those at highest risk may be reticent to engage with PrEP without community endorsement. Development of implementation strategies will necessitate an initial contextual review of community perceptions of PrEP and relevant education campaigns to balance messaging around HIV risk, PrEP use, and efficacy, keeping in mind the risk of stigmatizing the PrEP intervention.

Risk, and risk perception [41], have been extensively researched and debated in regards to translating into effective HIV prevention uptake [42–44]. Research published from the FEMPrEP efficacy trial found that women in the study who did not maintain PrEP use also did not accurately assess their own risk [45]. In the TAPS study, the primary results suggest that the population accessing PrEP are women who may be at self-acknowledged high risk, which was confirmed repeatedly through the IDIs [19]. However, it is unclear whether those who dropped out were accurately assessing their own risk, though one woman who dropped out of the PrEP arm early returned later with an HIV-positive result. The risks articulated in these IDIs, however, were directly linked to the dangers of sex work and closely intertwined with the acknowledgement of personal responsibilities. In addition, these personal responsibilities could also contribute to women’s abilities to maintain use if priorities for making money or going to school prevented them from making it to the clinic for visits and PrEP refills. At the same time, stress around condomless sex with main partners seemed to be reduced for some women who worried they might be at risk when partner fidelity was unclear.

While sex work can be a choice and many women have been empowered in their engagement in it [46], women in this sample of sex workers overwhelmingly felt that they currently had no options to transition out. Most transition programmes have failed because they don’t offer women options to make enough money to meet their perceived responsibilities [47]. In these IDIs, women had differing ideas about how to move on to other employment, with some asking for jobs in the clinic and others pursuing educations with the hope of empowering themselves to advance.

The notions of empowerment and control were underlying currents throughout the findings of these IDIs. While most of their worries remained constant throughout the progression of the interviews, there was also an alleviation of stress which occurred as PrEP efficacy became a personal reality. While PrEP is not a magic solution to all women’s problems, for some in this study it started to symbolize hope and future prospects, as well as peace of mind where safety was concerned, where women could view their business as business without as much fear, and sexual interactions with partners were less worrisome.

Interestingly, the findings in this paper at the thematic level are very similar to what has been published so far from qualitative research of PrEP use among MSM. Research published from an open-label PrEP study (following the iPrEX efficacy trial) described relief from stress around the possibility of HIV infection and a sense of security as additional benefits of using PrEP [48]. These feelings did not seem to change sexual behaviour, which from analyses conducted thus far and what has been reported in our qualitative research, is also what happened in TAPS [19]. Similar findings regarding the alleviation of fear around HIV were presented from another study of MSM in the United States [49]. Encouragingly, this research suggests that PrEP use may provide sources of critical, constructive, and empowering HIV protection for those at highest risk of HIV without subsequent risk compensation, as was widely feared [48,50–53].

Not as many women returned for second and third IDIs as hoped, which is a risk with any longitudinal research and in particular with more transient populations such as sex workers. It is difficult to assess why participants drop out over time when they do not provide specific reasons themselves. However, based on what we learned here and what was learned in earlier research preparing for the TAPS study [25,26], it is likely that a combination of social dynamics around belief in PrEP and its efficacy (including stigmatization of taking ARVs as a sign of illness), personal risk assessment which may fluctuate over time, personal responsibilities which supersede clinic visits and participation in research, and potential issues with substance use probably contributed to retention. For these reasons and to further unpack the dropout seen in TAPS to help support PrEP programming, additional research is being considered to potentially interview women who were lost to follow up, to determine the reasons.

Despite the limited number of second and third round IDIs, the process of serial interviews enabled us to examine progression of thinking and feeling around PrEP use. In most cases, the participants became more committed over time, and discovered personal strength in their commitment, even if adherence was not always perfect. Their willingness to be more open about issues with product use grew over time, which was a benefit of this method. The practicalities of use were not the central themes for any of the IDI participants, however, women were able to share how they managed adherence or cycled off and on PrEP over time. One prominent component of managing PrEP use, which became more apparent over time, was the development of supportive structures for pill taking. Some women found support from partners, but many more had family members or friends who were already HIV-positive and could provide inside knowledge of pill taking habits. In fact, the high prevalence of HIV in the community, and particularly among other women in sex work, could be another contributing factor for staying HIV-negative and a motivation for PrEP use. These notions reinforce the idea that synergies can be found among HIV-negative and positive populations, which may also help to destigmatise interventions.

### Limitations

The study ended up only enrolling just over half the originally planned sample size, which therefore limited the range of perspectives possible to include. Additionally, we planned to capture perspectives of women using PrEP as well as those cycling off, however those women who cycled off rarely stayed in the study. The study sites were also limited to urban settings, therefore findings may not translate to other contexts. While these are clear limitations of this research, it should also be noted that the interviews did reach saturation of data, where personal stories did vary but themes were consistent across FSW accounts.

## Conclusion

PrEP represents a valuable new HIV prevention option for women in sex work who are able to remain in care. Risks and responsibilities were the main expressed drivers of PrEP uptake and use in these interviews, however, personal and community disbelief in the reality of PrEP efficacy challenged this. As PrEP programming is scaled-up, it will be important to ensure accurate, relevant, and widespread messaging is employed in communities to generate demand and support for PrEP use, even if use is to be focused for those at highest risk. PrEP could be one of the most significant game-changers in HIV prevention for the past 20 years, and requires this kind of in-depth, qualitative research to understand if and how it impacts on women’s lives and what motivates them to take it up and adhere and thus how services can be better designed to respond to these needs.
